# Cyanoacrylate for Intraoral Wound Closure: A Possibility?

**DOI:** 10.1155/2015/165428

**Published:** 2015-11-15

**Authors:** Parimala Sagar, Kavitha Prasad, R. M. Lalitha, Krishnappa Ranganath

**Affiliations:** Department of Oral & Maxillofacial Surgery, Faculty of Dental Sciences, MSRUAS, MSRIT Post, MSR Nagar, Bangalore 560054, India

## Abstract

Wound closure is a part of any surgical procedure and the objective of laceration repair or incision closure is to approximate the edges of a wound so that natural healing process may occur. Over the years new biomaterials have been discovered as an alternate to conventional suture materials. Cyanoacrylate bioadhesives are one among them. They carry the advantages of rapid application, patient comfort, resistance to infection, hemostatic properties, and no suture removal anxiety. Hence this study was undertaken to study the effect of long chain cyanoacrylate as an adhesive for intraoral wound closure and also to explore its hemostatic and antibacterial effects. Isoamyl-2-cyanoacrylate (AMCRYLATE) was used as the adhesive in the study. In conclusion isoamyl cyanoacrylate can be used for intraoral wound closure, as an alternative to sutures for gluing the mucoperiosteum to bone, for example, after impaction removal, periapical surgeries, and cleft repair. Its hemostatic and antibacterial activity has to be further evaluated.

## 1. Introduction

Wound closure is a part of any surgical procedure. The objective of laceration repair or incision closure is to approximate the edges of a wound so that natural healing process may occur. To promote healing one should achieve precise wound approximation, reduction of patient discomfort, easy handling of the working properties of wound closure materials, and low infection rates [1].

There has been a search for new biomaterials over a few decades to serve as an alternate to conventional suture materials. Conventional suture materials have disadvantages like scar formation, need for dressing to protect the wound and suture, and revisit for suture removal [1]. One among the various biomaterials developed is the cyanoacrylate group. Cyanoacrylates have been found to be a good alternative to sutures in extraoral wound closure, as they carry advantages of rapid application, patient comfort, resistance to infection, hemostatic properties, and no suture removal anxiety [2].

**Scheme 1:**
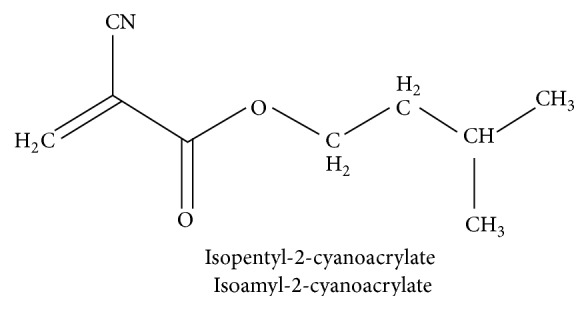
Structure of isoamyl-2-cyanoacrylate.

Cyanoacrylates were first recognized to have adhesive properties by Coover in 1959. Their general formula is CNCH_2_=COO-R where R is side chain [2] (Scheme 1).

They belong to the family of polymers whose monomer is formed by reversible condensation of formaldehyde with a cyanoacrylate ester. These adhesives polymerize in the presence of anions especially hydroxyl ions. This indicates that it forms a firm adhesive bond on coming in contact with water or tissue moisture, by undergoing exothermic polymerization. The number of alkyl groups in the side chain of cyanoacrylate can be increased from one (methyl cyanoacrylate), to two (ethyl), to four (butyl), and to five (isoamyl) but usually not more than eight (octyl cyanoacrylate). Isoamyl-2-cyanoacrylate contains 5 carbon alkyl constituents of the carboxyl group (COO-R).

These adhesives differ physically to meet the defined applications. The main distinguishing feature between the esters is the size of molecule. Methyl, the original cyanoacrylate ester, is the smallest. Consequently, a large number of polymer chains can be formed resulting in bonds with high tensile strength and also molecules, which are less toxic [3]. As these long chain acrylates degrade at a slower rate, they permit the degradation products to be more safely metabolized resulting in less intense inflammatory response.

Cyanoacrylates are maintained in a liquid state by an acidic stabilizer, which has the action of inhibiting the molecules from cross-linking. Partially ionized molecules of water, normally found on all surfaces exposed to the atmosphere, have the action of neutralizing the inhibitor. When applied on tissue surface the inhibitor is eliminated and the chemical action allows the molecule to polymerize in 10 seconds. The adhesive strength depends on relation to the approximation of the molecules attraction to each other. Physical locking is also a factor by virtue of the penetration of the adhesive into irregularities of the tissue surfaces [3].

Cyanoacrylates are biodegradable and their rate of removal from the application site is by polymer degradation and surface sloughing. They undergo hydrolytic attack of carbon-carbon bond to produce formaldehyde and cyanoacetate.

Wide applications have been cited in the literature from sealing of CSF leaks encountered during orbital surgery and repair of tympanic membrane to microvascular anastomosis [2]. Very few studies have been done about the intraoral use of cyanoacrylates. This study was undertaken to explore the various uses of cyanoacrylates intraorally.

In the present study isoamyl-2-cyanoacrylate was used after various surgical procedures intraorally. Isoamyl-2-cyanoacrylate was the only cyanoacrylate, which could be used intraorally and cost-effective when compared to others. Octyl cyanoacrylate could not be used as it is not indicated in areas subject to frequent moisture; hence isoamyl-n-cyanoacrylate was chosen for the study [4].

The aim of this study was to explore the various uses of isoamyl cyanoacrylates intraorally and also to study its hemostatic and antimicrobial effects.

## 2. Materials and Methods

The study conducted was a prospective observational in vivo study of patients attending the Department of Oral & Maxillofacial Surgery for various surgical procedures. Isoamyl-2-cyanoacrylate was used for wound closure. It was available from CONCORD DRUGS LIMITED under the trade name of “AMCRYLATE,” manufactured at Chennai, India.

10 patients reported to the Department of Oral & Maxillofacial Surgery at M. S. Ramaiah.

Dental College & Hospital for various surgical procedures from November 2005 to August 2007 were selected for the study. Isoamyl-2-cyanoacrylate was used intraorally for various indications/applications. Data was recorded according to the pro forma. After obtaining clearance from ethics committee, study was begun. Patient selection was based on the following inclusion and exclusion criteria. The study included patients with fracture mandible, cleft palate, mandibular third molar impaction, laceration, periapical cysts, and need for biopsy. Patients with infected wounds, patients with wounds where tension-free closure was not possible, and medically compromised patients were excluded from the study.

Cases selected for the study were to glue the mucoperiosteum to the underlying bone, following impacted mandibular 3rd molar removal, cases of periapical surgeries, or cases to glue periosteum to the palate in cleft patients. It was also used for gluing of collagen over the surgical wound, closure following incisional biopsy in case of carcinoma of alveolus, pyogenic granuloma, and gluing of a lacerated wound.

After completion of the procedure, AMCRYLATE available as (0.25 mL or 0.5 mL) ampule was loaded in a syringe provided along with the adhesive. The incised margins were then checked for approximation. Cyanoacrylate loaded in the syringe was applied over the bone and inner surface of the mucoperiosteal flap and also applied over the incised margins and then approximated into the desired position. It was held there under pressure for a few seconds till the polymerization process was complete to ensure firm adhesion of the flap to the underlying bone. Wound was observed on the 1st, 7th, and 15th postoperative day for presence/absence of inflammation, edema, approximation, or dehiscence. The findings were recorded and assessment was done based on the results.

## 3. Case Details and Results

A total of 10 patients were enrolled in the study and results tabulated (Table 1).

In case 1, a case of pleomorphic adenoma of palate located at the junction of hard and soft palate surgical excision was done. Overlying mucosa was also excised along with the tumor. Here cyanoacrylate was used to glue collagen membrane over the surgical wound to promote healing. On the 2nd postoperative day collagen membrane got dislodged; however, wound later healed without any complications.

**Figure 1:**
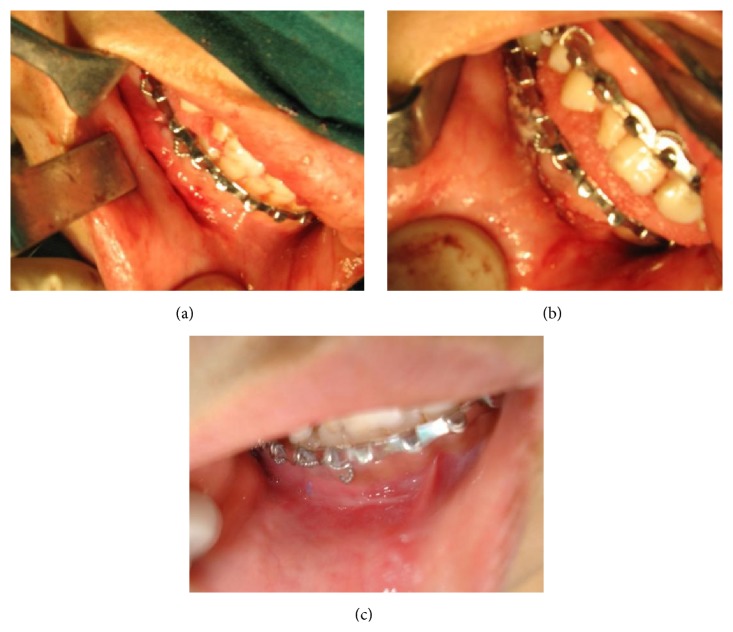
(a) Incision after submucosal closure. (b) Cyanoacrylate application over incision line. (c) Seventh postoperative day.

Case 2 was a patient with fracture of left angle and right parasymphysis of mandible. Here in the parasymphysis region fracture site was exposed, reduced, and fixed with titanium plates and screws. Submucosal suturing with VICRYL (Figure 1(a)) was done for layered closure; mucosal adhesion was achieved with cyanoacrylate (Figure 1(b)). Postoperative healing (Figure 1(c)) was uneventful.

In case 3, following incisional biopsy in case of carcinoma of left alveolus, cyanoacrylate was used to seal the biopsy site. The tissue was very fragile and suturing could have resulted in further trauma. Hemostasis was readily achieved.

In case 4, cyanoacrylate was used to cover the mucosal incision line over the bone graft in addition to sutures for airtight closure. This was a case of fracture of angle of mandible on right side. ORIF under GA was done, using titanium plates and screws. Postoperatively after one month patient returned back with nonhealing of fracture due to broken titanium plate. So fracture was fixed again with reconstruction plate over the angle region and iliac bone graft was used to promote healing as the defect was wide. Mattress suture was given intraorally over the mucosal surface. On the first postoperative day intraoral wound at the third molar region showed slight gaping at the graft site. So cyanoacrylate was used over the incision line, after, approximating the flap to cover the bone graft. Wound healing was successfully achieved later without any complication.

In cases 5 and 7, periapical surgery was performed for curetting apical cysts of maxillary anterior. Triangular shape flap was designed. Full thickness mucoperiosteum flap was reflected. After the surgical procedure mucoperiosteum flap was glued back to the underlying bone in its position with AMCRYLATE. In case 5 intraoral sinus discharging pus was present at the surgical site on the 15th postoperative day. Case 7 did not come back for review.

In case 6, cyanoacrylate was used to glue the mucoperiosteum and close the incision line (Ward's incision) after coronectomy of mandibular third molar (48) was done. No complications were noted and wound healed uneventfully.

In case 8, it was also used as a hemostatic agent in a patient with an exophytic growth over the maxillary canine-premolar region where biopsy was planned initially. Hemostasis could not be achieved in this case so complete excision was then done.

Case 9 was a patient with the midline cleft of the palate. Cyanoacrylate was used to affix the mucoperiosteum to the hard palate. The nasal mucosal layer was closed with VICRYL sutures beginning from uvula to alveolar ridges. Oral mucosal layer was then closed in the same manner. Instead of placing sling suture, to hold the mucoperiosteum to the underlying bone, cyanoacrylate was spread over mucoperiosteal flap and also on the hard palate. Gentle pressure for 1 min was applied over the hard palate to affix the flap. Postoperative wound healing was uneventful.

**Figure 2:**
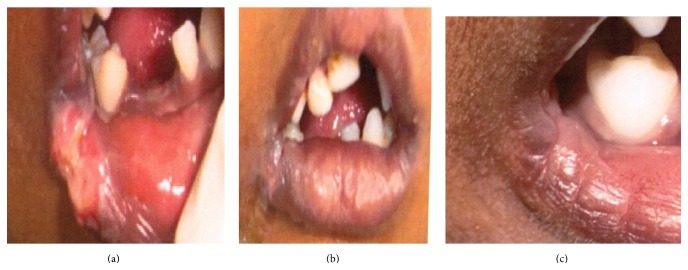
(a) Lacerated wound on lower lip. (b) Wound glued with cyanoacrylate. (c) 15th postoperative day.

Case 10 was a 4-year-old boy who presented with laceration at lip commissure (Figure 2(a)). Suturing was done under local anesthesia at the initial appointment. Wound gaped on the 2nd postoperative day. Cyanoacrylate was then used to glue the lacerated margin (Figure 2(b)), but again wound gaping was seen the next day. Cyanoacrylate was reapplied to the lacerated margins and dressing was given to protect the lacerated site. Wound later healed (Figure 2(c)) without complications.

*Summary*. Marginal seal/wound seal was acceptable as the incised margins were in close apposition with each other. Inflammation and edema were not present in maximum number of cases at the 15th postoperative day. One case of pus discharge was recorded after 15 days of surgery and one case of wound dehiscence was noted and gluing of the collagen membrane to the surgical site was not successful.

## 4. Discussion

The aim of all wound closure techniques is to approximate the edges of the wound so that natural process of uneventful healing takes place. In wound closure the primary focus should be on relieving tension on the wound and bringing the tissue edges together. Precise approximation of the incised/lacerated margins is critical to favorable cosmetic and functional results. The most commonly used method for closing lacerations is suturing [1]. This is the classic method of wound closure which carries the advantages of careful closure, good tissue approximation, highest resilient strength, and lowest dehiscence rate. However it has several disadvantages like requirement of anesthesia, greatest tissue reactivity, higher cost, more time consuming, need for suture removal, and associated risk of needle stick injury [1]. Over the past 3 decades new biomaterials have been discovered as an alternative to conventional suture material. These include staples, adhesive tapes, and tissue adhesives.

Cyanoacrylates include short chain (methyl and ethyl cyanoacrylates) and longer chain (butyl, isobutyl, isoamyl, and octyl cyanoacrylates) derivatives [2]. The long chain derivatives are the least histotoxic. Some authors have reported the clinical use of cyanoacrylates with several advantages including the ease of use with shorter operative time, formation of protective barrier, and painless application [2, 5]. The major disadvantage is reduced tensile strength (they should not be used in high tension areas) [5]. They also should be avoided over areas subject to frequent moisture and friction such as the hands and feet (unless splinted and dressed appropriately) [5]. These adhesives are contraindicated in infected wounds, immunocompromised patients, and patients with known allergy to cyanoacrylates and formaldehyde [5].

After discovery of cyanoacrylates in 1949, there has been interest in their use for various surgical procedures. These include traumatic laceration repair, bronchopleural fistula repair, repair of myocardial tears, mesh fixation for inguinal hernia repair, cosmetic rhinoplasty, embolization of intracranial AV malformations, and sealing of CSF leaks [6]. After use of these adhesives in various tissue organs it was found to be a valuable adjunct in intraoperative management of difficult problems and no case of general or local toxic reaction to this material was observed [6]. Initially it was used as an alternative to sutures only in superficial small and tension-free skin incisions or lacerations extraorally [7]. Now it has wide applications intraorally, for example, for promoting of healing in extraction sockets [8], as local hemostatic agent in warfarin treated patients undergoing oral surgery [9], for promoting of healing of recurrent aphthous ulcers [10], in cleft palate surgery [2], and for closing sinus membrane perforations during sinus lifts [11].

Numerous studies have been conducted on extraoral uses of cyanoacrylates in oral and maxillofacial region. Octyl cyanoacrylate was used to close neck incisions in cancer patients and has been found to be an effective alternate to sutures [12]. Another comparative study between isoamyl cyanoacrylate and butyl cyanoacrylate on healing of surgical incisions over the neck was done and isoamyl cyanoacrylate was found to be better than butyl acrylate [13]. However, very few studies have been conducted on intraoral applications of these cyanoacrylates. So this study was undertaken to explore the various uses of these acrylates intraorally. Animal studies on intraoral application of cyanoacrylates have been successful and are safe and suitable without any significant differences in liver and kidney functions [14]. In the present study, it was found that, in cases 5 and 7, cyanoacrylate could be successfully used in closure of mucoperiosteum in periapical surgery (triangular), in case 6, after impaction removal (Ward's incision), and, in case 9, for cleft palate closure (gluing mucoperiosteum). Results obtained in cleft patient are comparable to the results obtained in a previous study where butyl cyanoacrylate was used for gluing mucoperiosteum to palate [2]. Further comparative studies between the use of sutures and cyanoacrylate adhesive for mucoperiosteal closure in a larger sample of patients can clearly establish its usefulness. In our study cyanoacrylate was used as adjuvant in closing the mucosa in fracture cases as in cases 2 and 4. In case 2, wherein patient had fracture parasymphysis mucosal closure with acrylate was done after submucosal closure with sutures. In case 4 it was used to cover the bone graft site intraorally, in addition to sutures at the angle region of mandible. In both the cases successful closure was achieved with acrylate. Further comparative studies with a larger sample need to be conducted so that it replaces sutures successfully in closing the incision lines intraorally.

In a study conducted in the year 2003, butyl cyanoacrylate was effectively used as local hemostatic agent; warfarin treated patients underwent extraction of teeth without a change in their anticoagulation level [9]. In the present study, hemostasis was successfully achieved in case 3, when used at biopsy site of one patient (later reported as squamous cell carcinoma of left buccal mucosa), but was unsuccessful in case 8. In case 8, patient with exophytic growth in the maxilla, acrylate was applied over the bleeding site; though mucosal seal was obtained, bleeding continued submucosally. Hemostasis was achieved only after complete excision of the lesion. It was reported as pyogenic granuloma. Its use as a local hemostatic agent therefore needs further evaluation. Collagen glued to the underlying surgical site in case 1 (at the junction of hard and soft palate) got dislodged on the 2nd postoperative day. This may be due to friction created by food materials while eating and swallowing. Hence, cyanoacrylate in such situations can be used as an adjuvant to sutures rather than alternate to sutures. Use of cyanoacrylate will help in adhesion to the underlying arched (curved) palate as seen in case 9. Wound dehiscence was seen on the second day in the 4-year-old kid, that is, case 10 with lip laceration at the angle of mouth. Sutures also were not successful in approximating the margins at this site. This may be due to patient being uncooperative/unable to follow instructions. Also constant movement of the sutured/glued site during opening and closing of mouth and excessive moisture due to drooling of saliva could have been further hindrances. So use of adhesives in this region is comparable to sutures. However the second application of cyanoacrylate resulted in wound closure and better patient compliance with cyanoacrylate was seen as it was painless and less time consuming.

In our study group 9 patients had no significant signs of infection at the 15th postoperative day. Pus discharge was recorded in one case with periapical surgery after 15 days. Symptomatic treatment with antibiotics and analgesics was done and patient reviewed after a week. Sinus opening in relation to canine and first premolar was still persistent. Vitality test and IOPA radiograph confirmed nonvital first premolar. RCT was done and later wound healed successfully. So probably pus discharge may not be related as reaction to cyanoacrylate but due to nonvital first premolar. Inflammatory edema seen in all cases on the first postoperative day can be related to surgical trauma. Successful mucosal wound seal in all cases was obtained.

Future of tissue adhesives can be as broad as imagination. New ways to manufacture these agents more safely are being explored by better defining the synthetic additions. The biodegradable adhesives can be engineered for slow release of medications as delivery of medications to a specific target site is very desirable [4]. Closure of wounds without the need for sutures will be a major advancement, an opportunity to improve care for patients, especially children, and reduce pain and anxiety caused by the treatment.

## 5. Conclusion

Isoamyl-2-cyanoacrylate tissue adhesive was equivalent to suture and other traditional epidermal wound closure devices in providing adequate wound healing after closure of surgical incisions. There are several advantages of these adhesives; some of them are as follows: they have easy and painless application, need to remove sutures is eliminated, also they act as a hemostatic agent, and most important is better patient cooperation compared to sutures for the procedures done under local anesthesia. Cyanoacrylates can be used as an alternative to sutures for gluing the mucoperiosteum to bone, for example, after impaction removal, periapical surgeries, cleft closure, or biopsy. It can be used as an adjuvant to sutures in many situations to offer wound protection and reduce dehiscence and infection rates, for example, at bone graft sites, for skin grafts or collagen retention, and for mucosal closure at fracture sites. Its hemostatic, antimicrobial, and analgesic properties require further evaluation. Its cost-effectiveness is still debatable but it is an adhesive worth considering for intraoral closure. Further randomized clinical trials should definitely highlight its distinction from sutures and help in evolving a set of guidelines for intraoral use so as to achieve maximum results of this revolutionary product.

## Figures and Tables

**Table 1:** 

Results tabulation												
	Inflammation			Edema			Dehiscence			Wound seal/closure		
	1st POD	7th POD	15th POD	1st POD	7th POD	15th POD	1st POD	7th POD	15th POD	1st POD	7th POD	15th POD
Case 1	√	×	×	√	×	×	×	×	×	√	×	×
Case 2	√	×	×	√	×	×	×	×	×	√	√	√
Case 3	√	×	×	√	×	×	×	×	×	√	√	√
Case 4	√	×	×	√	×	×	×	×	×	√	√	√
Case 5	√	×	×	√	×	×	×	×	×	√	√	√
Case 6	√	×	×	√	×	×	×	×	×	√	√	√
Case 7	√	×	×	√	×	×	×	×	×	√	√	√
Case 8	—	—	—	—	—	—	—	—	—	—	—	—
Case 9	√	×	×	√	×	×	×	×	×	√	√	√
Case 10	√	×	×	√	×	×	√	×	×	×	√	√

×: absent, √: present, and —: could not be assessed.

Case 1: case of pleomorphic adenoma.

Case 2: fracture of right parasymphysis of mandible. Mucosal closure done.

Case 3: case of carcinoma of left alveolus. Hemostasis achieved at biopsy site with acrylic.

Case 4: case of fracture of right angle of mandible. Acrylate used to cover the incision line over the bone graft.

Case 5: case of periapical cyst. Mucoperiosteum glued with acrylate.

Case 6: case of impacted 48. Ward's incision closed with acrylate.

Case 7: case of periapical cyst. Mucoperiosteum glued with acrylate.

Case 8: case of pyogenic granuloma. Acrylate used to achieve hemostasis.

Case 9: case of cleft palate. Mucoperiosteum glued with acrylate.

Case 10: case of laceration of angle of mouth. Wound glued with acrylate.
